# Epidemiological Characteristics and Their Influencing Factors Among Pulmonary Tuberculosis Patients With and Without Diabetes Mellitus: A Survey Study From Drug Resistance Surveillance in East China

**DOI:** 10.3389/fpubh.2021.777000

**Published:** 2022-01-24

**Authors:** Qian Wu, Min Wang, Yu Zhang, Wei Wang, Teng-Fei Ye, Kui Liu, Song-Hua Chen

**Affiliations:** ^1^Department of Tuberculosis Control and Prevention, Zhejiang Provincial Center for Disease Control and Prevention, Hangzhou, China; ^2^Quzhou City Center Blood Station, Quzhou, China; ^3^Anhui No.2 Provincial People's Hospital, Hefei, China

**Keywords:** tuberculosis, diabetes mellitus, risk factor, drug resistance, poly-drug resistant

## Abstract

**Background:**

The burden of pulmonary tuberculosis (TB) and diabetes mellitus (DM) have become serious global concerns, while the comprehensive evaluations of DM status and drug resistance in TB patients are still lacking.

**Methods:**

All details of TB cases were collected from drug resistance monitoring sentinels in Zhejiang province. Fisher's exact test or Pearson chi-square test (χ^2^) was used to compare the baseline characteristics among TB with different DM statuses. The logistic regression model was used to estimate the relationship between DM and different drug resistance spectra. Univariate analysis and multivariate logistic model were used to explore the possible risk factors of drug resistance in TB patients with DM and no DM.

**Results:**

936 TB cases with smear-positive in Zhejiang province were collected, in which 76 patients (8.12%) owned the co-morbidity of DM. TB-DM prevalence was higher in older, Han nationality, employed, accompanied by no health insurance and hepatitis B status. Among 860 cases of TB-no DM and 76 cases of TB-DM, drug resistance-TB accounted for 31.51% and 23.68% (*P* > 0.05), MR-TB accounted for 15.93% and 14.47% (*P* > 0.05), respectively. MDR-TB was 4.88% and 6.58% (*P* > 0.05). The incidence of poly-drug resistant tuberculosis (PDR-TB) in TB-no DM patients (10.70 vs. 2.63%, OR: 4.43; 95% CI, 1.07–18.36) was higher than that in the TB-DM group (*P* < 0.05). In univariate and multivariate analysis, none of the basic factors were statistically significant with drug resistance among TB-DM cases (all *P* > 0.05). Retreatment was the risk factor of drug resistance among TB-no DM cases.

**Conclusions:**

Our results showed that the drug resistance rate of the TB-DM group was not higher than that of the TB-no DM group. Patients with TB-no DM were at a higher risk for PDR-TB, but not for MDR-TB, MR-TB, and drug resistance-TB. Special attention should be paid to TB-no DM patients who have been previously treated. In the future, large-scale and well-designed prospective studies are needed to clarify the impact of DM on the drug-resistance among TB.

## Background

Tuberculosis (TB) and diabetes mellitus (DM) are the top 10 causes of death globally, and the adverse effect of DM on TB has been widely investigated (1, 2). Although the mortality caused by pulmonary TB has undergone a progressive decline worldwide in recent decades, it is still a huge challenge in low- and middle-income countries (3). Also, with the population aging, nutrition transition, and obesity, the burden of DM is rising, including a 3-fold increased risk of developing TB (4, 5). Based on the global TB report released by the world health organization (WHO), China had the second-highest burden of TB. In Zhejiang province, there was a total of 29,576 active TB cases with the prevalence of 52.25/100,000 only in 2018 (6). Meanwhile, the age-standardized incidence of Type 2 DM, in Zhejiang Province, was released to be 281.73 (95% CI: 281.26–282.20) per 100,000 person-years, 293.19 (95% CI: 292.51–293.87) in males and 270.42 (95% CI: 269.76–271.09) in females during 2007–2017 (5). Thus, the burden of TB and DM, including the co-morbidity of both, was heavy in Zhejiang province.

To our knowledge, drug resistance emerges as a challenge to global TB control, and some known factors such as prior TB treatment, intravenous drugs, and DM might cause unfavorable effects on TB treatment (7). For DM, one study in Mexico implied a more than 3.5 times greater risk of developing drug resistance (8). Moreover, it could significantly increase the risk of developing MDR-TB, and its influence enhances to 2.43 times after adjusting the confounding factor (9). Previous studies showed that DM caused a series of severe impacts on MDR-TB cases, including increasing the risk of treatment failure and relapse (10–14). DM patients were also more susceptible to INH-resistant TB than non-DM (15). However, there was still a paucity of relevant studies and data in east China.

In Zhejiang province of China, the drug resistance monitoring for TB started from 1999 to 2000, and it was carried out every 4–5 years. Given the heavy disease burden of TB and DM in China, this study was performed to assess the prevalence of drug resistance among TB in Zhejiang province, explore the profile of TB patients with different DM statuses, and identify the possible influencing factors of drug resistance among TB-DM and TB-no DM cases.

## Methods

### Setting and Participants

In Zhejiang province, we had randomly constructed 30 sentinels in designated hospitals from all 90 counties for pulmonary TB surveillance. We evaluated the prevalence of DM among smear-positive pulmonary TB using simple random sampling. There were a total of 784 pulmonary TB cases needed in our research based on the formula: *N*= (1.96/0.035)^2*^0.5^*^(1–0.5) = 784 with 95% precision and a margin of error of 3.5% (16). Given a possible loss rate of 10%, we tried to collect more than 900 smear-positive cases in all sentinels. From July 1, 2013 to June 31, 2014, each sentinel should recruit more than 30 sputum smear-positive pulmonary TB patients. Three sputum samples (instant sputum, night sputum, and next day morning sputum) of each case were collected, and patients with sputum smear-positive >2 were included in the study. Compared with the standard drug-resistant strains, a drug sensitivity test was carried out in the provincial reference laboratory. Besides, the united questionnaire was designed for all included cases, including baseline information such as age, sex, education level, family income, and clinical information such as treatment history, symptoms, and DM status. The DM information of all included cases should be checked in Basic Public Health Information Systems (BPHIS) and Diabetes and Cancer Surveillance System (DCSS) of Zhejiang Province in China (17). All included pulmonary TB cases should complete the questionnaire. For patients under 14 years old or mentally disabled, the questionnaire was completed by their guardians and/or parents. Additionally, telephone surveys, family surveys, and consulting family members as feasible methods were allowed in our study. All data entry was completed by both clinician and disease control personnel by way of double-entry. To avoid error, project staff from the Center for Disease Control and Prevention (CDC) of Zhejiang Province checked the information in the end.

### Laboratory Diagnosis and Drug Susceptibility Testing

Acid-fast bacillus (AFB) smear microscope was carried out directly in the laboratory of the designated hospital in each county. Additionally, solid Löwenstein-Jensen (L-J) culture was used to obtain pulmonary TB strain. Identification of pulmonary TB strain and performing drug susceptibility testing (DST) was confirmed in Zhejiang provincial reference laboratory. DST was performed on L-J medium by the proportional method. All drugs were tested by the same method and compared with the results of standard strains. Drug concentration for rifampicin (RMP), ethambutol (EMB), isoniazid (INH), streptomycin (SM), kanamycin (KM), ofloxacin (OFX), levofloxacin (LVFX), moxifloxacin (MOX), capreomycin (CPM), amikacin (AM), para-aminosalicylic acid (PAS), cycloserine (CS), protionamide (PTO) were 40.0, 2.0, 0.2, 4.0, 30.0, 2.0, 2.0, 0.25, 40.0, 40.0, 1.0, 40.0, and 40.0 μg/mL, respectively. The details of the design and approach have been described in our previous study (16). The diagnosis of DM in BPHIS and DCSS refers to the guidelines for the prevention and treatment of type 2 DM in China (2010 Edition), including fasting plasma glucose (FPG) and oral glucose tolerance test (OGTT) (18).

### Definitions

Initial treatment cases were defined as cases that had never previously been treated for pulmonary TB or had taken anti-TB drugs for <1 month. Previously treated cases were defined as cases that had received 1 month or more of anti-TB drugs in the past. Drug resistance-TB was defined as having acquired or primary drug resistance based on the history of previous treatment. Pulmonary TB patients with or without DM have been termed TB-DM and TB-no DM, respectively. Monoresistance (MR) represents resistance to only one anti-TB drug. Polydrug resistance (PDR) is defined as resistance to more than one anti-TB drug, in addition to isoniazid and rifampicin. Multidrug resistance (MDR) refers to pulmonary TB resistance to at least the two major anti-tuberculosis drugs isoniazid, and rifampicin (19). History of pulmonary TB contact was defined as having been in close contact with smear-positive TB cases for more than 8 h or smear-negative TB cases for more than 40 h in the past 3 months (20). Pulmonary TB symptoms include cough for more than 2 weeks, hemoptysis, and cough for 1–2 weeks, along with any of the above symptoms (21). According to the China Statistical Yearbook Year 2015, low income in our study refers to those patients whose annual income is less than RMB 10,877 (22).

### Ethics Statement

Our study was approved by the Ethics Committee of the Zhejiang Provincial Center for Disease Control and Prevention (ZJCDC). All participants signed the unified informed consent, and all personal information was kept confidential as required.

### Statistical Analysis

Baseline characteristics of pulmonary TB case classification included age stratification, sex (male, female), ethnic group (Han nationality, others), residence (urban, rural), employment (employed/other, unemployed), education (none/primary school, junior school, senior school, and above), health insurance (yes, no), Low income (yes, no), hepatitis B (yes, no), type (initial treatment, retreatment), history of pulmonary TB contact (yes, no) and pulmonary TB symptoms (yes, no) was compared between cases with and without DM. When the expected frequency was >5 and the sample size was >40 in the contingency table, the Pearson chi-square test was used. When the expected frequency was <5 but >1 in the contingency table with the same sample size above, continuity correction for the Pearson chi-square test was used. Otherwise, Fisher's exact test was applied. Univariate analysis and multivariate logistic models were used to estimate the relationship between DM and different drug resistance profiles (e.g., drug resistance-TB, MR-TB, MDR-TB, PDR-TB). Similarly, the logistic regression model was used to explore the risk factors of drug resistance in TB-DM patients. Based on previous studies, the covariates were adjusted by the multivariate model (23). All statistical analysis was performed by R software (version 3.5.3). Bilateral *P* < 0.05 was considered to be statistically significant.

## Results

### Baseline Characteristics

During the study period, 936 TB patients' drug sensitivity data and DM-related information were included; 860 cases (91.88%) were TB-no DM patients, 76 cases (8.12%) were TB-DM patients. The average age of TB-no DM patients (44.58 ± 20.30) was less than that of TB-DM patients (56.55 ± 14.42) years (*P* < 0.01). The age distribution of TB-no DM cases and TB-DM patients: under 14 years old accounted for 0.47% and 1.32%, 15–24 years old accounted for 19.42% and 0%, 25–44 years old accounted for 36.63% and 14.47%, 45–64 years old accounted for 22.67% and 59.21%, and over 65 years old accounted for 20.81 and 25.00%. The proportion of male cases was 69.53% (598/860) and 76.32% (58/76), respectively (*P* > 0.05). The proportion of Han nationality cases was 89.65% and 98.68% (*P* < 0.05). The proportion of rural cases was 76.98 and 73.68%, respectively (*P* > 0.05). The proportion of employed/other persons in the two groups were 77.44 and 90.79%, respectively (*P* < 0.01). The educational level of none/primary school among TB-no DM cases and TB-DM patients were 21.40 and 27.63%, with junior school 60.58 and 59.21%, and senior school and above 18.02 and 13.16%, respectively (*P* > 0.05). The proportion of TB-DM patients without health insurance (96.05%) was higher than that of TB-no DM patients (78.49%) (*P* < 0.01). The proportion of low-income people with TB-DM was 25.00% while that of TB-no DM patients was 18.49% (*P* > 0.05). The results showed that TB-DM patients were more likely to have hepatitis B (7.89 vs. 2.33%) (*P* < 0.01). There was no significant difference between TB-no DM group and TB-DM group in retreatment type (9.65 vs. 14.47%), TB contact history (6.98 vs. 3.95%) and TB symptoms (96.63 vs. 93.42%) (*P* > 0.05) (Table 1).

**Table 1:** Baseline characteristics of tuberculosis (TB) cases with and without diabetes mellitus (DM).

**Factors**	**TB-no DM patients (%)**		**TB-DM patients (%)**		***P*-value**
	**(*n* = 860)**		**(*n* = 76)**		
**Age (years)**					
Average	44.58 ± 20.30		56.55 ± 14.42		<0.01^**^
0–14	4	(0.47)	1	(1.32)	
15–24	167	(19.42)	0	(0.00)	
25–44	315	(36.63)	11	(14.47)	
45–64	195	(22.67)	45	(59.21)	
≥65	179	(20.81)	19	(25.00)	
**Sex**					0.22
Male	598	(69.53)	58	(76.32)	
Female	262	(30.47)	18	(23.68)	
**Ethnic group**					0.01
Han nationality	771	(89.65)	75	(98.68)	
Others	89	(10.35)	1	(1.32)	
**Residence**					0.52
Urban	198	(23.02)	20	(26.32)	
Rural	662	(76.98)	56	(73.68)	
**Employment**					0.01
Employed/other	666	(77.44)	69	(90.79)	
Unemployed	194	(22.56)	7	(9.21)	
**Education**					0.33
None/primary school	184	(21.40)	21	(27.63)	
Junior school	521	(60.58)	45	(59.21)	
Senior school and above	155	(18.02)	10	(13.16)	
**Health insurance**					<0.01
Yes	185	(21.51)	3	(3.95)	
No	675	(78.49)	73	(96.05)	
**Low income** ^**a**^					0.09
Yes	159	(18.49)	19	(25.00)	
No	671	(78.02)	50	(65.79)	
**Hepatitis B**					0.01^*^
Yes	20	(2.33)	6	(7.89)	
No	840	(97.67)	70	(92.11)	
**Diagnostic Type**					0.18
Initial treatment	777	(90.35)	65	(85.53)	
Retreatment	83	(9.65)	11	(14.47)	
**History of TB contact**					0.31
Yes	60	(06.98)	3	(3.95)	
No	800	(93.02)	73	(96.05)	
**TB symptoms**					0.27^*^
Yes	831	(96.63)	71	(93.42)	
No	29	(3.37)	5	(6.58)	

a *Low income data available for 899 patients*.

* *For results of Pearson chi-square test with continuity correction*.

** *For results of Fisher's exact test*.

### Drug-Resistance Profile

As presented in Table 2, the DST results of all patients were obtained in this study. Among 860 TB-no DM patients and 76 TB-DM patients, drug resistance-TB accounted for 31.51% and 23.68% (*P* > 0.05), and MR-TB accounted for 15.93% and 14.47% (*P* > 0.05), respectively. Compared with susceptible strains, the OR and its 95% CI of PDR among DM or not was 4.43 (95%CI: 1.07–18.36, *P* = 0.03), while no significance was identified among subgroups of MDR, MR-TB, and drug resistance-TB, respectively (*P* > 0.05).

**Table 2:** Drug-resistance profile of diagnosed tuberculosis (TB) cases with and without diabetes mellitus (DM).

**Drug resistance**	**TB-no DM patients (%)**		**TB-DM patients (%)**		**OR (CI 95%)**	***P***
	**(*n* = 860)**		**(*n* = 76)**			
Drug resistance-TB (total)	271	(31.51)	18	(23.68)	1.48 (0.86, 2.57)	0.16
**Any drug resistance**						
INH	109	(12.67)	8	(10.53)	1.23 (0.58–2.64)	0.59
RFP	55	(6.40)	5	(6.58)	0.97 (0.38–2.50)	0.95^*^
EMB	27	(3.14)	2	(2.63)	1.20 (0.28–5.14)	0.81^*^
SM	131	(15.23)	12	(15.79)	0.96(0.50–1.82)	0.90
OFX	41	(4.77)	3	(3.95)	1.22 (0.37–4.03)	0.97^*^
AK	3	(0.35)	0	(0.00)	1.00	0.61^**^
KM	9	(1.05)	0	(0.00)	1.00	0.37^**^
PAS	13	(1.51)	0	(0.00)	1.00	0.28^**^
CPM	16	(1.86)	0	(0.00)	1.00	0.23^**^
CS	28	(3.26)	1	(1.32)	2.52 (0.34–18.81)	0.56^*^
PTO	61	(7.09)	3	(3.95)	1.86 (0.57–6.07)	0.30
LVFX	34	(3.95)	2	(2.63)	1.52 (0.36–6.47)	0.79^*^
MOX	24	(2.79)	0	(0.00)	1.00	0.14^**^
**MR-TB**	137	(15.93)	11	(14.47)	1.12 (0.58–2.18)	0.74
INH	22	(2.56)	2	(2.63)		
RFP	10	(1.16)	0	(0.00)		
EMB	6	(0.70)	1	(1.32)		
SM	60	(6.98)	7	(9.21)		
OFX	1	(0.12)	0	(0.00)		
KM	1	(0.12)	0	(0.00)		
CPM	6	(0.70)	0	(0.00)		
CS	9	(1.05)	0	(0.00)		
PTO	22	(2.56)	1	(1.32)		
**MDR**	42	(4.88)	5	(6.58)	0.73 (0.28–1.90)	0.71^*^
MDR1: RFP+INH	10	(1.16)	0	(0.00)		
MDR2: RFP + INH + EMB + SM	3	(0.35)	0	(0.00)		
MDR3: RFP + INH + SM	11	(1.28)	2	(2.63)		
Others	18	(2.09)	3	(3.95)		
**PDR**	92	(10.70)	2	(2.63)	4.43 (1.07–18.36)	0.03
PDR1: INH + EMB	0	(0.00)	0	(0.00)		
PDR2: INH + SM	16	(1.86)	1	(1.32)		
PDR3: RFP + SM	0	(0.00)	0	(0.00)		
Others	76	(8.84)	1	(1.32)		

* *For results of Pearson chi-square test with continuity correction*.

** *For results of Fisher's exact test*.

### Risk Factors of Drug Resistance Among TB-DM Cases

Among 76 TB-DM patients with DST results, 58 (76.32%) were fully susceptible and 18 (23.68%) were drug-resistant. In univariable analysis, we found that none of these factors, including age (45–64 years, OR: 1.22, 95% CI: 0.28–5.23; or ≥65 years, OR: 0.35, 95% CI: 0.05–2.51), sex (female, OR: 0.90, 95% CI, 0.25–3.18), residence (rural, OR: 0.91, 95% CI: 0.28–2.97), employment (unemployed, OR: 1.33, 95% CI: 0.23–7.49), education (junior school, OR: 1.37, 95% CI, 0.38–4.96; senior school and above, OR: 1.82, 95% CI: 0.32–10.34), health insurance (no, OR: 0.61, 95% CI: 0.05–7.11), low income (no, OR: 1.87, 95% CI: 0.47–7.49), hepatitis B (no, OR: 1.60, 95% CI: 0.17–14.70) and type (retreatment, OR: 2.08, 0.53–8.14), was statistically significant with drug resistance (*P* > 0.05). Likewise, the results of the multivariable analysis also showed that there were no statistically significant risk factors for drug resistance among TB-DM cases (*P* > 0.05) (Table 3).

**Table 3:** Risk factors of drug resistance among TB-DM cases.

**Factors**	**Non-drug resistance**		**Drug resistance-TB**		**Univariable analysis**	***P*-value**	**Multivariable analysis**	***P*-value**
	**(*n* = 58)**		**(*n* = 18)**		**OR (95% CI)**		**OR (95% CI)**	
**Age (years)**								
0–44	9	(15.52)	3	(16.67)	Reference		Reference	
45–64	32	(55.17)	13	(72.22)	1.22 (0.28–5.23)	0.79	3.27 (0.48–22.35)	0.23
≥65	17	(29.31)	2	(11.11)	0.35 (0.05–2.51)	0.30	0.52 (0.03–8.09)	0.64
**Sex**								
Male	44	(75.86)	14	(77.78)	Reference		Reference	
Female	14	(24.14)	4	(22.22)	0.90 (0.25–3.18)	0.87	1.75 (0.36–8.39)	0.49
**Ethnic group**								
Han nationality	57	(98.28)	18	(100.00)	Reference		Reference	
Others	1	(1.72)	0	(0.00)		1.00		1.00
**Residence**								
Urban	15	(25.86)	5	(27.78)	Reference		Reference	
Rural	43	(74.14)	13	(72.22)	0.91 (0.28–2.97)	0.87	0.77 (0.16–3.71)	0.75
**Employment**								
Employed/other	53	(91.38)	16	(88.89)	Reference		Reference	
Unemployed	5	(8.62)	2	(11.11)	1.33 (0.23–7.49)	0.75	0.23 (0.02–3.34)	0.28
**Education**								
None/primary school	17	(29.31)	4	(22.22)	Reference		Reference	
Junior school	34	(58.62)	11	(61.11)	1.38 (0.38–4.96)	0.63	1.52 (0.30–7.59)	0.61
Senior school and above	7	(12.07)	3	(16.67)	1.82 (0.32–10.34)	0.50	1.39 (0.11–18.21)	0.80
**Health insurance**								
Yes	56	(96.55)	17	(94.44)	Reference		Reference	
No	2	(3.45)	1	(5.56)	0.61 (0.05–7.11)	0.69	0.44 (0.02–9.28)	0.60
**Low income**								
Yes	16	(27.59)	3	(16.67)	Reference		Reference	
No	37	(63.79)	13	(72.22)	1.87 (0.47–7.49)	0.37	1.84 (0.32–10.55)	0.49
**Hepatitis B**								
Yes	5	(8.62)	1	(5.56)	Reference		Reference	
No	53	(91.38)	17	(94.44)	1.60 (0.17–14.70)	0.68	3.72 (0.34–41.10)	0.28
**Type**								
Initial treatment	51	(87.93)	14	(77.78)	Reference		Reference	
Retreatment	7	(12.07)	4	(22.22)	2.08 (0.53–8.14)	0.29	2.27 (0.41–12.70)	0.35
**History of TB contact**								
Yes	3	(5.17)	0	(0.00)	Reference		Reference	
No	55	(94.83)	18	(100.00)		1.00		1.00
**TB symptoms**								
Yes	53	(91.38)	18	(100.00)	Reference		Reference	
No	5	(8.62)	0	(0.00)		1.00		1.00

### Risk Factors of Drug Resistance Among TB-No DM Cases

Among 860 TB-no DM patients with DST results, 589 (68.49%) were fully susceptible and 271 (31.51%) were drug-resistant. In univariable analysis, we found that type (retreatment, OR: 2.08, 1.32–3.29) was the risk factor of drug resistance among TB-no DM cases. However, age (45–64 years, OR: 1.14, 95% CI: 0.80–1.63; or ≥65 years, OR: 1.10, 95% CI: 0.76–1.58), sex (female, OR: 0.89, 95% CI, 0.65–1.22), residence (rural, OR: 1.08, 95% CI: 0.76–1.52), employment (unemployed, OR: 1.09, 95% CI: 0.78–1.54), education (junior school, OR: 0.98, 95% CI, 0.68–1.41; senior school and above, OR: 1.07, 95% CI: 0.67–1.68), health insurance (no, OR: 0.86, 95% CI: 0.61–1.22), low income (no, OR: 0.95, 95% CI: 0.66–1.38), hepatitis B (no, OR: 0.56, 95% CI: 0.23–1.35), history of TB contact (no, OR: 1.56, 95% CI: 0.84–2.89) and TB symptoms (no, OR:1.34, 95% CI: 0.63–2.88) was not statistically significant with drug resistance (*P* > 0.05). Consistently, the results of the multivariable analysis also showed that type (retreatment, OR: 2.09, 1.30–3.35) was a significant risk factor for drug resistance among TB-no DM cases (Table 4).

**Table 4:** Risk factors of drug resistance among TB-no DM cases.

**Factors**	**Non-drug resistance**		**Drug resistance-TB**		**Univariable analysis**	***P*-value**	**Multivariable analysis**	***P*-value**
	**(*n* = 589)**		**(*n* = 271)**		**OR (95% CI)**		**OR (95% CI)**	
**Age (years)**								
0–44	338	(57.39)	148	(54.61)	Reference		Reference	
45–64	130	(22.07)	65	(23.99)	1.14 (0.80, 1.63)	0.46	1.17 (0.78, 1.75)	0.45
≥65	121	(20.54)	58	(21.4)	1.11 (0.76, 1.58)	0.63	1.26 (0.78, 2.05)	0.35
**Sex**								
Male	405	(68.76)	193	(71.22)	Reference		Reference	
Female	184	(31.24)	78	(28.78)	0.89 (0.65, 1.22)	0.47	0.94 (0.67, 1.32)	0.74
**Ethnic group**								
Han nationality	524	(88.96)	247	(91.14)	Reference		Reference	
Others	65	(11.04)	24	(8.86)	0.78 (0.48, 1.28)	0.33	0.86 (0.51, 1.45)	0.56
**Residence**								
Urban	138	(23.43)	60	(22.14)	Reference		Reference	
Rural	451	(76.57)	211	(77.86)	1.08 (0.76, 1.52)	0.68	1.13 (0.77, 1.64)	0.54
**Employment**								
Employed/other	459	(77.93)	207	(76.38)	Reference		Reference	
Unemployed	130	(22.07)	64	(23.62)	1.09 (0.78, 1.54)	0.62	1.07 (0.73, 1.57)	0.72
**Education**								
None/primary school	126	(21.39)	58	(21.40)	Reference		Reference	
Junior school	359	(60.95)	162	(59.78)	0.98 (0.68, 1.41)	0.91	1.01 (0.66, 1.54)	0.97
Senior school and above	104	(17.66)	51	(18.82)	1.07 (0.67, 1.68)	0.79	1.25 (0.72, 2.16)	0.43
**Health insurance**								
Yes	122	(20.71)	63	(23.25)	Reference		Reference	
No	467	(79.29)	208	(76.75)	0.86 (0.61, 1.22)	0.40	0.82 (0.56, 1.19)	0.29
**Low income**								
Yes	108	(18.34)	51	(18.82)	Reference		Reference	
No	463	(78.61)	208	(76.75)	0.95 (0.66, 1.38)	0.79	1.07 (0.71, 1.60)	0.76
**Hepatitis B**								
Yes	11	(1.87)	9	(3.32)	Reference		Reference	
No	578	(98.13)	262	(96.68)	0.56 (0.23, 1.35)	0.20	0.63 (0.24, 1.62)	0.34
**Type**								
Initial treatment	545	(92.53)	232	(85.61)	Reference		Reference	
Retreatment	44	(7.47)	39	(14.39)	2.08 (1.32, 3.29)	0.00	2.09 (1.30, 3.35)	0.00
**History of TB contact**								
Yes	46	(7.81)	14	(5.17)	Reference		Reference	
No	543	(92.19)	257	(94.83)	1.56 (0.84, 2.89)	0.16	1.48 (0.78, 2.83)	0.24
**TB symptoms**								
Yes	571	(96.94)	260	(95.94)	Reference		Reference	
No	18	(3.06)	11	(4.06)	1.34 (0.63, 2.88)	0.45	1.18 (0.52, 2.65)	0.69

## Discussion

Our results provide the epidemiological characteristics of DM in patients with TB. We observed that the TB-DM ratio among TB cases in the Zhejiang province of China was 8.12%. One meta-analysis of 84 studies from 31 low-income and middle-income countries illuminated that the prevalence of DM in TB patients ranged from 1.80 to 45%, and the majority was between 10 and 30% (24). Though our result was relatively lower than the existing report, the ratio might increase sharply with the increasing aging population. Literatures indicates that chronic diseases such as DM are essential factors that damage the host's resistance to TB (25), and TB-DM cases need more robust TB treatment and follow-up (26). According to a modeling study, it estimates that in the next 20 years, preventing the rise of DM will avert 6 million TB cases (95% CI: 5.1–6.9) in 13 high TB burden countries, and a combined response need to be constructed to meet the challenge of TB and DM (27).

Our data suggest that TB-DM patients are more likely to be older, Han nationality, employed, accompanied by no health insurance and hepatitis B than TB-no DM cases. Age may be a coexisting risk factor for TB and DM. Besides, older people are susceptible to chronic diseases. It suggests that early screening for DM in elderly TB patients should be strengthened. Similar to the effect factor of the elderly, decreased host defense in patients with hepatitis B may increase the risk of active TB infection caused by *Mycobacterium tuberculosis* strains (28). Compared with TB-no DM patients, the proportion of TB-DM co-morbidity patients among the employed population is significantly higher. It is strongly recommended to screen for active TB among DM patients in the occupational population. The health insurance rate of people with TB-DM is relatively low, probably because most people without health insurance have low incomes. It further suggested that economic status may be a risk factor for TB-DM co-morbidity, and more health focus such as health education and health promotion should be considered for these specific groups.

Our results provide the limited difference in drug resistance profiles between TB-DM and TB-no DM patients. Previous studies revealed that DM is associated with an increased risk of any various acquired drug resistance, including drug resistance TB and MDR-TB (29–31). On the contrary, the observation of TB cases in Zhejiang province showed that DM was not a risk factor for drug resistance. The inconsistency of these conclusions may be due to the limited study population and sampling method. Further results indicated that TB-no DM patients were at a higher risk for PDR-TB, but not for MDR-TB, MR-TB, and drug resistance-TB. Although possible reasons caused this finding not to be explored and verified in our study, it might be attributed to patients with TB-no DM being more likely to be infected with PDR. Consistent with our results, a systematic search found no significant impact of DM on the MDR or rifampicin resistance TB (32). However, some retrospective cohort studies have found that DM is significantly associated with MDR-TB (OR: 2.26, 95% CI: 1.66–3.07), Asian (OR: 1.40, 95% CI: 1.01–1.95) subgroups (33), and in Georgia (adjusted OR 2.27, 95%CI 1.02–5.08) (34). These contradictory findings highlight the need to conduct large-scale and well-designed prospective studies. Furthermore, a number of studies have evaluated risk factors associated with DM in TB patients, including age, sex, BMI, and household contact history with TB (30, 31, 35). However, our study's results from univariable and multivariable regression analysis did not support any significant risk factors comparing TB-DM patients with and without drug-resistant TB. Retreatment of TB involves recurrence, failure, and poor compliance of patients with previous treatments (36). In China, drug resistance among retreatment TB cases remain a concerning public health issue. Acquired drug resistance is usually found among retreatment TB cases. A meta-analysis in Iran showed that the prevalence of any drug resistance in previously treated patients (65.6%) was significantly higher than in new TB patients (23.0%) (37). In the univariable analysis and multivariable analysis, we found that retreatment was the risk factor of drug resistance among TB-no DM cases. Therefore, our study indicates that special attention should be paid to TB-no DM patients who were previously treated.

Several limitations of the present study should be addressed. First, the diagnosis of DM is based on the previous medical records of PTB patients, which was not verified in our design. In addition, the number of patients in groups is limited, which might lead to the possibility of information deviation. Despite these limitations, this study has some strengths. The total number of drug resistance-TB patients is divided into different subgroups, such as MDR-TB, PDR-TB, MR-TB, SM-resistant TB, and INH-resistant TB, *etc*., which would enable us to explore the relationship between DM and TB strains with different antibiotic-resistant mutations. Secondly, the known second-line anti-TB drug tested by DST had also been considered in this study.

## Conclusions

In summary, TB-DM patients are prone to be older, Han nationality, employed, accompanied by no health insurance, and hepatitis B. The drug resistance rate of the TB-DM group was not higher than that of TB-no DM group. Patients with TB-no DM were at a higher risk for PDR-TB, but not for MDR-TB, MR-TB, and drug resistance-TB. Special attention should be paid to TB-no DM patients who were previously treated. In the future, large-scale and well-designed prospective studies are needed to clarify the impact of DM on the drug-resistance among TB.

## Data Availability Statement

The raw data supporting the conclusions of this article will be made available by the authors, without undue reservation.

## Ethics Statement

The studies involving human participants were reviewed and approved by Zhejiang Provincial Center for Disease Control and Prevention. The patients/participants provided their written informed consent to participate in this study.

## Institutional Review Board Statement

Our research was approved by the Ethics Committee of Zhejiang Center for Disease Control and prevention. Before data collection, statistical analysis and reporting, the monitoring station will delete any personal identification number of TB patients. All patients obtained informed consent and the study was conducted according to the Helsinki declaration.

## Author Contributions

QW, KL, and S-HC conceptualized the study, participated in the study design, and revised the manuscript. QW wrote the manuscript and conducted the statistical analysis. MW, YZ, WW, and T-FY collected the data or provided the revised advice. All authors contributed to the article and approved the submitted version.

## Funding

This study was supported by NHC Key Laboratory of Health Technology Assessment (Fudan University, FHTA2019-05), Philosophy and Social Science Project of Zhejiang Province (19NDJC243YB), and Zhejiang Provincial Medical and Health Project (2021KY618 and 2019RC135).

## Conflict of Interest

The authors declare that the research was conducted in the absence of any commercial or financial relationships that could be construed as a potential conflict of interest.

## Publisher's Note

All claims expressed in this article are solely those of the authors and do not necessarily represent those of their affiliated organizations, or those of the publisher, the editors and the reviewers. Any product that may be evaluated in this article, or claim that may be made by its manufacturer, is not guaranteed or endorsed by the publisher.

## Abbreviations

TB: tuberculosis
DM: diabetes
DST: drug susceptibility test
MR-TB: mono-resistant tuberculosis
DR-TB: drug-resistant tuberculosis
MDR-TB: Multidrug-resistant tuberculosis
PDR-TB: poly-drug resistant tuberculosis
RMP: rifampin
INH: isoniazid
SM: streptomycin
EMB: ethambutol
OFX: ofloxacin
LVFX: levofloxacin
MOX: moxifloxacin
CPM: capreomycin
CS: cycloserine
KM: kanamycin
PAS: para-aminosalicylic acid
PTO: protionamide
AM: amikacin.
